# Retrospective analysis of fractionated intensity-modulated radiotherapy (IMRT) in the interdisciplinary management of primary optic nerve sheath meningiomas

**DOI:** 10.1186/s13014-019-1438-2

**Published:** 2019-12-27

**Authors:** Franziska Eckert, Kerstin Clasen, Carina Kelbsch, Felix Tonagel, Benjamin Bender, Ghazaleh Tabatabai, Daniel Zips, Daniela Thorwarth, Bettina Frey, Gerd Becker, Helmut Wilhelm, Frank Paulsen

**Affiliations:** 1 Department of Radiation Oncology, Eberhard-Karls-University Tuebingen, Hoppe-Seyler-Str. 3, 72076 Tuebingen, Germany; 2 Centre for Neurooncology, Eberhard-Karls-University Tuebingen, Hoppe-Seyler-Str. 3, 72076 Tuebingen, Germany; 3 Department for Ophthalmology, Eberhard-Karls-University Tuebingen, Elfriede-Aulhorn-Str. 7, 72076 Tuebingen, Germany; 4 Department of Diagnostic and Interventional Neuroradiology, Eberhard-Karls-University Tuebingen, Hoppe-Seyler-Str. 3, 72076 Tuebingen, Germany; 5 Department of Radiation Oncology, Section for Biomedical Physics, Eberhard-Karls-University Tuebingen, Hoppe-Seyler-Str. 3, 72076 Tuebingen, Germany; 6 RadioChirurgicum, CyberKnife Suedwest, Klinik am Eichert, Eichertstr. 3, 73035 Goeppingen, Germany

**Keywords:** Meningioma, Optic nerve, Stereotatic radiotherapy, Neuroophthalmology, Visual field, Visual acuity

## Abstract

**Background:**

As optic nerve sheath meningiomas (ONSM) are rare, there are no prospective studies. Our retrospective analysis focusses on a cohort of patients with uniform disease characteristics all treated with the same radiotherapy regimen. We describe treatment decision making, radiotherapy planning and detailed neuro-ophthalmological outcome of the patients.

**Methods:**

26 patients with unilateral ONSM extending only to the orbit and the optic canal were evaluated for neuro-ophthalmological outcome. Radiation treatment was planned in a simultaneous integrated boost approach to gross tumor volume (GTV) + 2 mm / 5 mm to 54 Gy / 51 Gy in 1.8 Gy / 1.7 Gy fractions. Follow-up was done by specialized neuro-ophthalmologists. Visual acuity and visual field defects were evaluated after therapy as well as during follow-up.

**Results:**

Interdisciplinary treatment decision for patients with ONSM follows a rather complex decision tree. Radiation treatment planning (equivalent uniform dose (EUD), maximum dose to the optic nerve) improved with experience over time. With this patient selection visual acuity as well as visual field improved significantly at first follow-up after treatment. For visual acuity this also applied to patients with severe defects before treatment. Long term evaluation showed 16 patients with improved visual function, 6 were stable, in 4 patients visual function declined. Interdisciplinary case discussion rated the visual decline as radiation-associated in two patients.

**Conclusions:**

With stringent patient selection radiotherapy for unilateral primary ONSM to 51 Gy / 54 Gy is safe and leads to significantly improved visual function. Interdisciplinary treatment decision and experience of the radiation oncology team play a major role.

## Background

High precision radiotherapy has become an established treatment option for optic nerve sheath meningiomas (ONSM) [1–4]. Technical developments in radiation oncology with improved pretreatment imaging (anatomical [5] as well as functional [6–8] imaging), modern radiotherapy techniques such as intensity-modulated radiotherapy (IMRT) and volumetric arc radiotherapy (VMAT) as well as the availability of image-guided radiotherapy (IGRT) allow for the treatment of small target volumes in elaborate regions [9–11]. High-precision, image-guided radiotherapy is able to spare normal tissue and reduce side effects while increasing the dose to the target volume and thus local control. Simultaneous integrated boost concepts have been proposed to achieve high dose coverage to the target volume and decrease the risk of side effects [12–15].

For ONSM the main objective for patient management is the preservation of visual function as these benign tumors do not threaten patients’ survival and show slow growth and high local control rates after treatment [16]. Thus, treatment indication has to be evaluated carefully in an interdisciplinary setting of neuro-ophthalmologists and radiation oncologists [17, 18]. Likewise, for radiotherapy treatment planning, radiation dose to organs at risk needs to be strictly prioritized over dose coverage for the target volume.

There are no prospective outcome data for either surgery or radiotherapy for ONSM due to the rarity of the disease. Published case series mostly describe heterogeneous cases. Heterogeneity is based on either primary ONSM confined to the N. opticus and secondary ONSM with spreading of skull base meningiomas into the optic canal [3], different treatment strategies such as Gamma-knife radiosurgery and stereotactic fractionated radiotherapy [19] or photon and proton irradiation [20] with different dose and fractionation regimens or are limited to less than 10 patients [21–24]. However, with all these inconsistencies, local control and functional outcome after high-precision radiotherapy for ONSM seem to be promising and severe side effects and toxicity seem to be rare. Our own experience with 3D-conformal stereotactic radiotherapy showed improvement of visual acuity and a decrease in visual field deficits in approximately 10 and 30% of the patients, respectively [25–27].

Here, we report the interdisciplinary patient selection as well as the results of stereotactic, fractionated IMRT for a homogenous patient cohort with unilateral, primary ONSM, all treated with the same radiation regimen and planning objectives.

## Methods

This single institution retrospective analysis includes all ONSM patients treated with IMRT with simultaneous integrated boost for unilateral optic nerve sheath meningiomas limited to the orbit and optic canal from 2008 to 2017. Of 28 patients initially identified two were excluded from the analysis due to missing follow-up information. Estimated median follow-up was 2.2 ± 0.5 years, ranging from 3 months (short term visual outcome assessable) to 8.6 years. Detailed patient characteristics are given in Table 1. The project was approved by the local ethics committee (417/2017BO2).

**Table 1:** Patient characteristics

Age Median / range (years)	47.9	22.2–82.4
	*n*	*%*
Sex		
Male	8	31
Female	18	69
Side		
Right	16	62
Left	10	38
Time from diagnosis to radiotherapy		
< 1 year	22	85
> 1 year	4	15
PET for radiotherapy planning		
No	4	15
Yes	22	85
Growth pattern		
Sheathlike	18	69
Fusiform	8	31
Involvement of optic canal		
No	9	35
Yes	17	65
Use of corticosteroids		
No	16	62
Yes	10	38

All patients with suspected ONSM, e.g. loss of vision without pathological findings in the bulbus oculi or suspicious findings in MRI exams due to other symptoms, underwent detailed ophthalmological exams performed by specialized neuro-ophthalmologists. Work-up consisted of best corrected visual acuity, visual field testing (30° supraliminal automated static perimetry), pupil function, ocular motility, slitlamp examination, ophthalmoscopy (photography) and optical coherence tomography (OCT) to assess the intraretinal neural structures. Imaging consisted of gadolinium-enhanced MRI and positron emission tomography (PET)-CT / PET-MRI with somatostatin-receptor-analoga tracer in the majority of cases (22/26 patients).

For radiotherapy planning patients were immobilized with thermoplastic masks and a planning CT scan was acquired with 2 mm / 3 mm slice thickness. For contouring MRI and PET imaging was co-registered to the planning CT dataset. The gross tumor volume (GTV) was delineated using all available clinical and imaging information. Expansion of the GTV by 2 mm / 5 mm (3 mm / 6 mm craniocaudally) resulted in the planning target volume (PTV)54 / PTV51, respectively. Planning objectives were coverage of the PTVs by the 95% isodose of the prescribed dose of 51 Gy in 30 fractions (1.7 Gy / fraction) for PTV51 and 54 Gy in 30 fractions (1.8 Gy / fraction) for PTV54 in a simultaneous integrated boost concept. Concerning organs at risk, the maximal total dose to the optic nerve and optic chiasm was restricted to 54 Gy. Sparing of these organs at risk was prioritized over target volume coverage as long as the PTV was encompassed by the 90% isodose (Table 2). Treatment planning was performed using Hyperion®, a Monte-Carlo based planning algorithm using an equivalent uniform dose (EUD) concept. Treatment techniques consisted of step-and-shoot IMRT, sliding window IMRT or VMAT. Patient positioning during radiotherapy was verified by three-dimensional cone-beam CT imaging daily for the first three fractions. In case of patient shifts of less than 2 mm in all cone-beam CTs, frequency was reduced to weekly controls. Treatment was performed with a high precision linear accelerator with multileaf collimators (MLCs) of 4–5 mm (Elekta Agility, Crawley, UK).

**Table 2:** Planning objectives for radiotherapy of unilateral, intraorbital ONSM

Priority	Volume	Dose constraint	Minor deviation
1	Optic chiasm	Dmax < 54 Gy	
2	Optic nerve	Dmax < 54 Gy	Dmax < 54.2 Gy
3	PTV54	D98 > 95%	D98 > 90%
4	PTV51	D98 > 95%	D98 > 90%
5	Retina / eye	D2 < 40 Gy	D2 < 54 Gy

Follow-up ophthalmological examinations were done every three months with a complete neuro-ophthalmological workup. Gadolinium enhanced MR imaging was performed annually. Improval / decline of visual acuity was defined as a change of ≥0.2 log steps. Improval / decline of visual field was defined as a change of ≥10%. In case of discordant findings, the overall visual outcome was rated as stable. Functional outcome was evaluated three months after treatment as well as for latest available follow-up after radiotherapy.

The statistical analysis was performed with the software package SPSS 24 (SPSS Inc., Chicago, IL, USA). Values are given ± standard error. Means were compared by paired or unpaired student’s t-test provided that the assumptions of the test were met by the data. Medians were compared by non-parametric t-test. Correlation of continuous variables was tested by linear regression. Correlation of categorical variables was tested by Chi-square test. Follow-up was estimated by the Kaplan Meier method. Statistical significance was defined for a *p*-value < 0.05.

## Results

### Patient selection and indication for radiotherapy

As ONSM are benign tumors and are sometimes stable in size and symptoms over years, one crucial step in the treatment of these patients is the selection of patients to be treated and the timing of therapy. Patients with clinical suspicion of ONSM based on slow decline of vision or incidental imaging finding underwent detailed neuro-ophthalmological examination including best corrected visual acuity, visual field, pupil function, ocular motility, slitlamp, ophthalmoscopy (photography) and OCT. These findings were correlated with gadolinium-enhanced MR imaging. Typical imaging findings for ONSM include sheath-like contrast enhancement around the optic nerve or an intraorbital mass with close spatial relationship to the optic nerve and gadolinium enhancement. Typical ophthalmological findings were reduced visual acuity, relative afferent pupillary defect, visual field loss, normal, swollen or pale optic disc. In case of normal visual acuity and visual field, patients underwent active surveillance with three-monthly ophthalmological assessment and MRI follow-up. Patients with mild or moderate symptoms were only scheduled for treatment after documented progressive loss of vision. Radiotherapy was planned in cases with typical findings in somatostatin-receptor-analoga PET-CT. Typically, ONSM show high uptake of somatostatin receptor analogon compared to normal tissue (except for the hypophysis). In the case of atypical findings, biopsy was considered. For patients with severe symptoms, the integrity of retinal neural structures was assessed by OCT. OCT is helpful in treatment decision making, although interpretation may be difficult. Oedema of the nerve fibre layer can be misinterpreted as normal thickness of nerve fibre and ganglion cell layer although a considerable ganglion cell and axonal loss may be present. If nerve fibre and ganglion cell layer thickness is reduced irreversible optic nerve damage is confirmed. A careful interpretation of morphological findings and correlation with function and history is necessary.

**Fig. 1:**
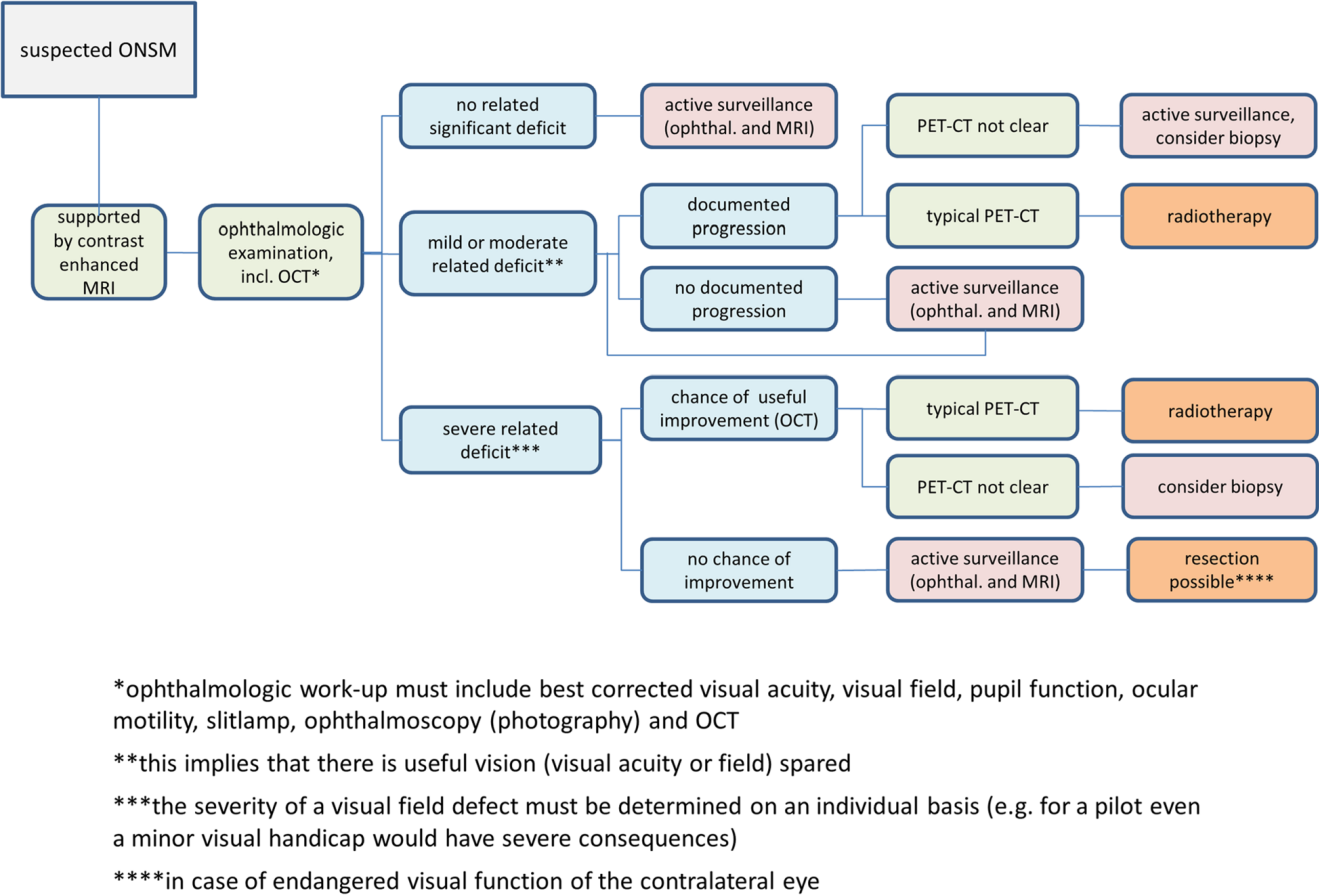
Flowchart of clinical management of patients with suspected OSNM. Not all patients with suspected ONSM need immediate intervention, such as radiotherapy. Ophthalmologic work-up must include best corrected visual acuity, visual field, pupil function, ocular motility, slitlamp, ophthalmoscopy (photography) and OCT. The complex algorithm for the management of patients indicates the necessity of close interdisciplinary cooperation

Absence of ganglion cell layer and nerve fibre loss usually led to the indication for radiotherapy, even in cases with very poor vision. Therapy was initiated in a timely manner because of the chance for recovering of visual function. Patients with neural degeneration and no useful visual function underwent active surveillance for the visual function of the contralateral eye and were scheduled for radiotherapy or surgery in the case of threatened or declining visual function contralaterally (Fig. 1).

In our patient cohort 22 patients (85%) had been diagnosed during the workup for substantial vision loss and were referred for radiotherapy after diagnosis. Three patients underwent active surveillance for 1.3 years, 2.5 years and 15 years respectively before undergoing radiotherapy following first signs of declining visual function. One patient had undergone three surgical procedures over 15 years prior to radiotherapy, which was started at progression of visual symptoms.

### Radiobiological considerations and radiation treatment planning

Equivalent dose in 2 Gy dose per fraction (EQD2) was calculated with the linear quadratic model for prescribed doses for optic nerve and PTV51 / PTV54. For tumor control (meningioma) α/β was considered to be 3.76 Gy [28]. Thus, the prescribed doses are equivalent to EQD2_3.76_ of 48.34 Gy and 52.13 Gy for PTV51 and PTV54, respectively. A maximum dose to the optic nerve of 54 Gy in 1.8 Gy fractions corresponds to 51.30 Gy EQD2_2_ with α/β = 2 Gy as described for the optic chiasm [29].

**Fig. 2:**
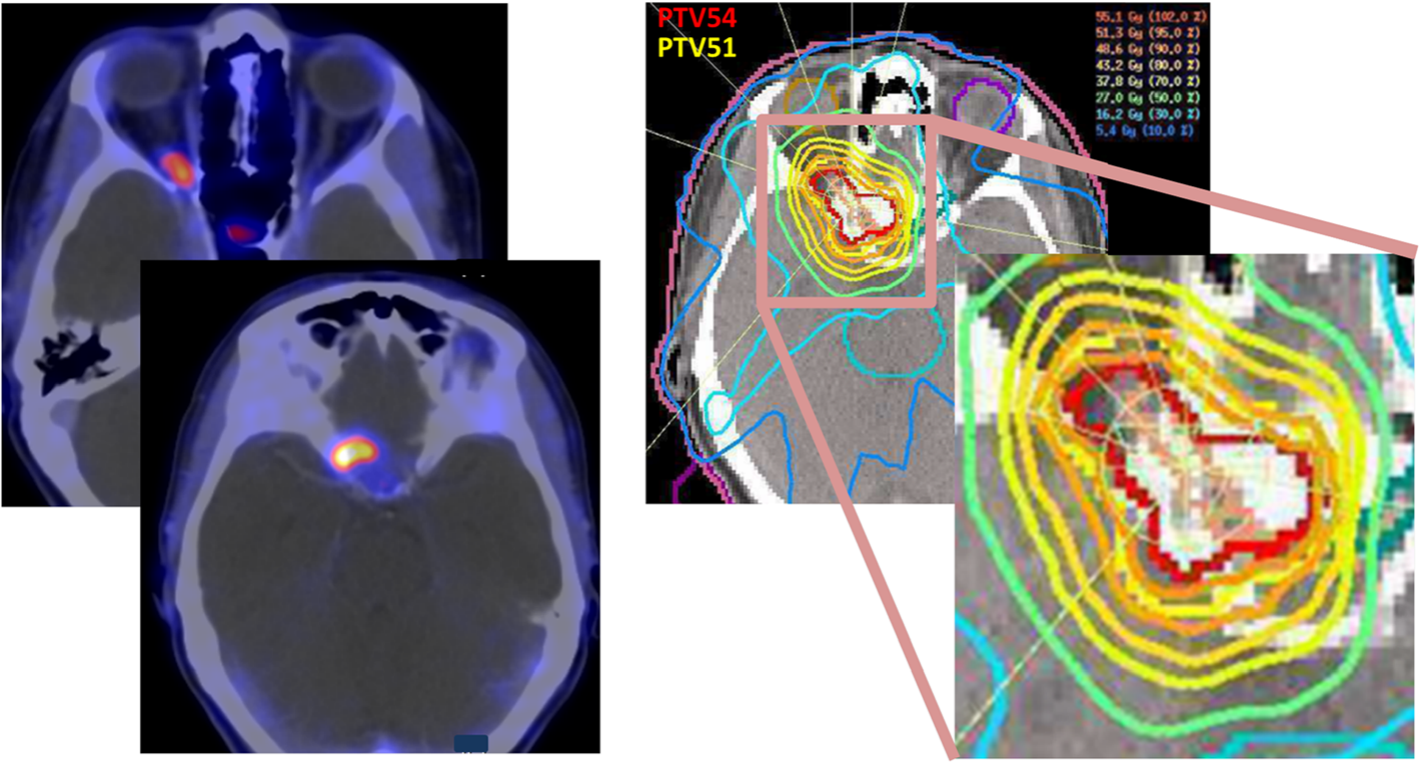
Typical example of a somatostatin-receptor-analogon PET-CT (68-Gallium-DOTATATE) with an average standard-uptake-value (SUV) of 4.8. Physiological uptake of the hypophysis can be seen in the upper image. The radiation plan shows steep dose gradients with sparing of the contralateral optic nerve

Mean target volumes for radiotherapy planning were 4.09 ± 0.70 cm^3^, 7.80 ± 1.06 cm^3^ and 15.78 ± 1.66 cm^3^ for GTV, PTV54 and PTV51, respectively. A typical example of PET findings and radiation plan is depicted in Fig. 2. As our prescribed dose of 54 Gy was rather high and to optimize functional outcome, the main planning objective was not to exceed the tolerance dose to the optic nerve or other organs at risk (Tbl. 2). Maximal physical dose to the optic nerve was 53.92 ± 0.05 Gy for all patients, corresponding to EQD2_2_ of 51.18 ± 0.07 Gy. D2 of the optic nerve did not exceed 54 Gy (physical dose) in any patient.

**Fig. 3:**
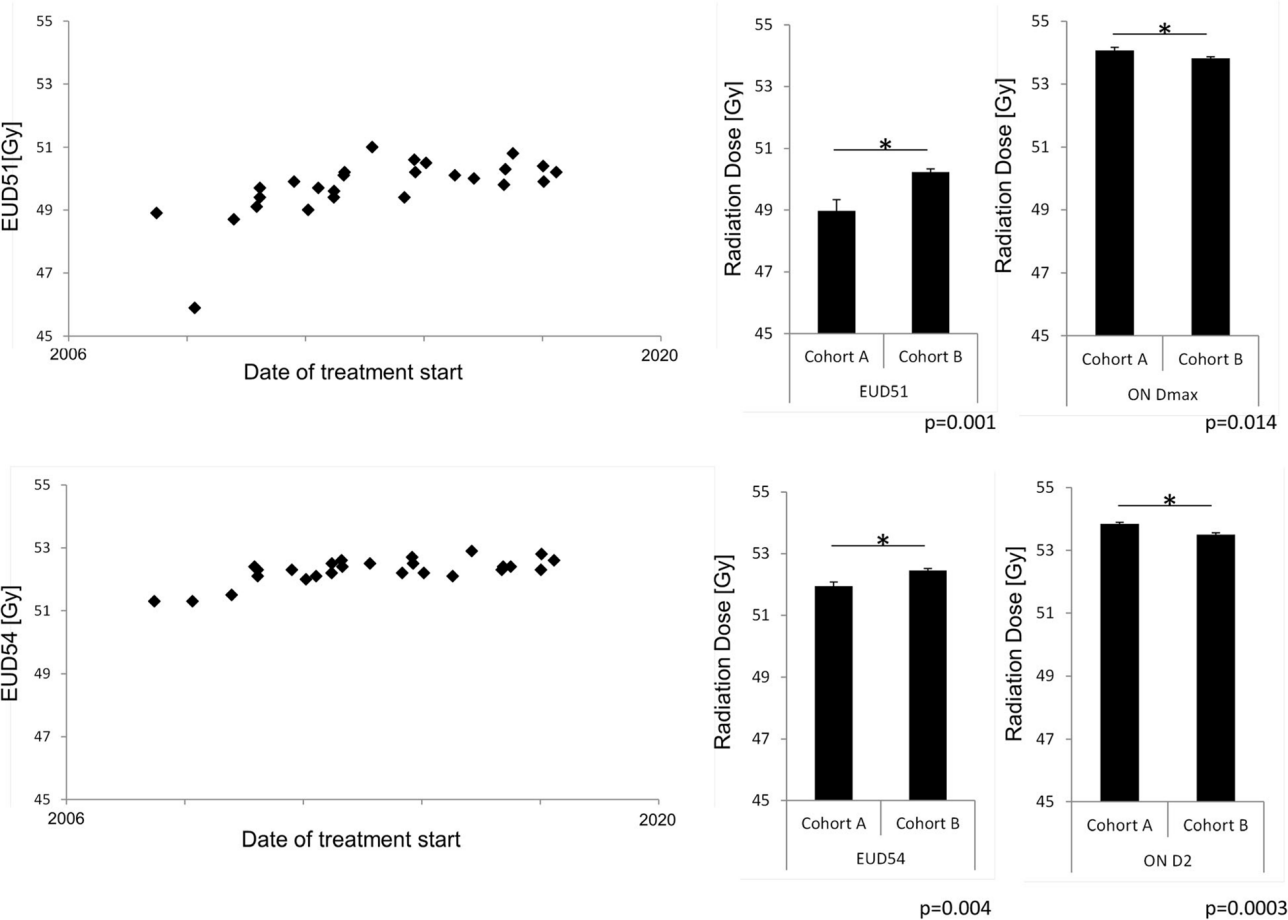
EUD plotted against the date of treatment start shows a typical learning curve with an initial increase followed by a plateau phase. EUD51 (EUD of PTV51) and EUD54 (EUD of PTV51) significantly increased from cohort A (first ten patients) to cohort B (remaining patients). Dmax as well as D2 to the optic nerve was significantly lower in cohort B compared to cohort A

As EUD of PTV51 and PTV54 was not used as planning objective, but might be a measure of plan quality and tumor control, we evaluated EUDs for both volumes. EUD was 52.27 ± 0.08 Gy, corresponding to 96.8% of the prescribed dose for PTV54 and 49.72 ± 0.19 Gy, corresponding to 97.5% of the prescribed dose for PTV51. As shown in Fig. 3, plotting EUD of PTV51 and PTV54 against start of treatment, resulted in increasing EUDs for the first patients, stabilizing after a learning curve. Comparison of EUDs for the 10 first treated patients (Cohort A) compared to the other 16 (Cohort B) showed highly significantly higher EUDs for patients treated after the learning curve (Fig. 3). At the same time, Dmax for the optic nerve was significantly lower in cohort B with 53.82 ± 0.04 Gy versus 54.07 ± 0.09 in cohort A (*p* = 0.01, data not shown).

### Short term visual function at first follow-up after radiotherapy

**Fig. 4:**
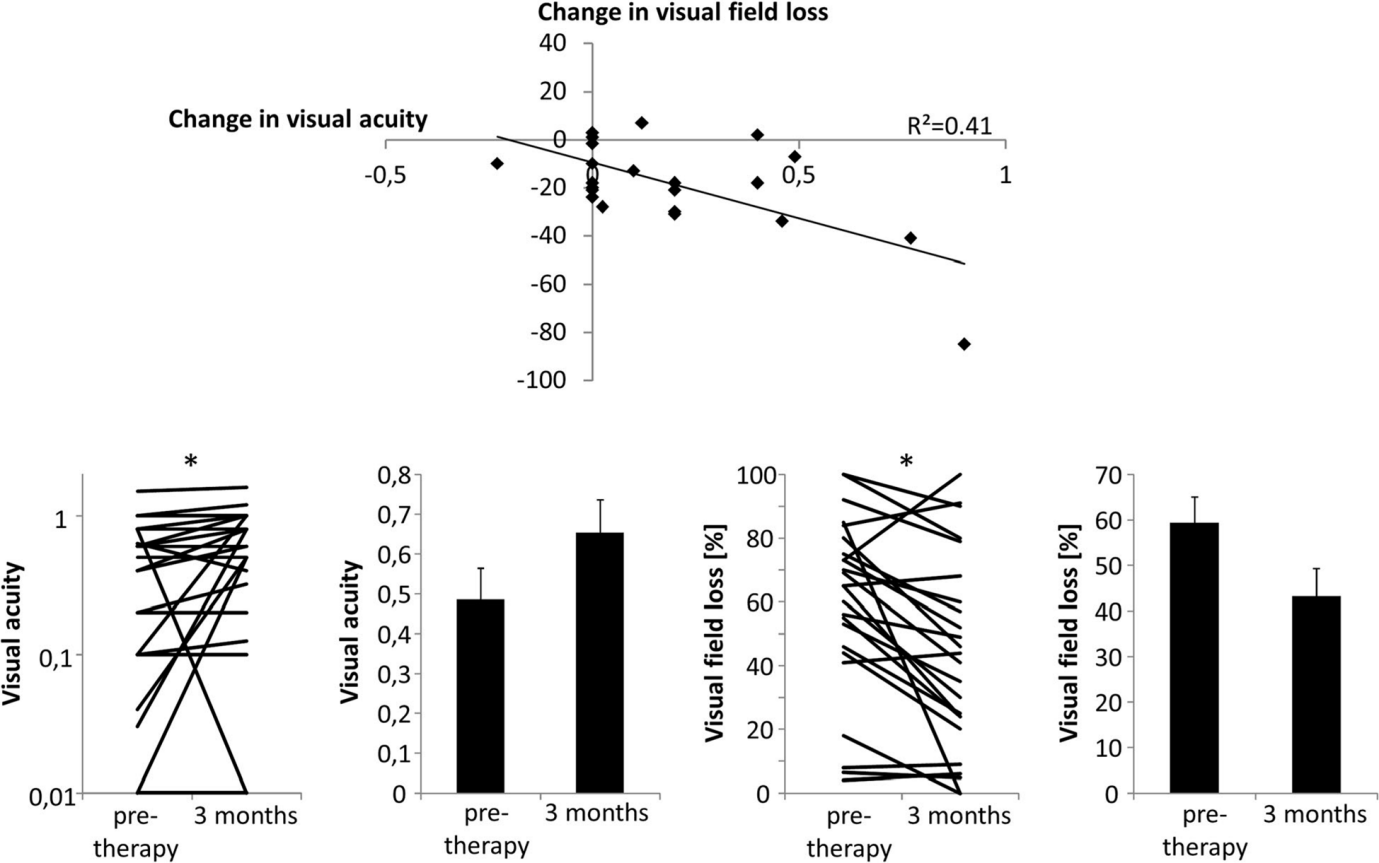
Short term ophthalmologic outcome was determined at first control visit after radiotherapy (up to 3 months after end of treatment). The changes in visual field and visual acuity only showed a moderate correlation. Visual acuity as well as visual field showed significant improval in the selected patients treated according to the algorithm depicted in Fig. 1. Notably, even patients with severe defects before the start of therapy showed improved function afterwards

At time of treatment start patients had significant functional ophthalmological deficits with a median visual acuity of 0.45 (0.01–1.5) and a median visual field loss of 65% (4–100%). These functional parameters improved significantly at first control (up to three months) after therapy to 0.70 (0.01–1.6; *p* = 0.04) and 42.5% (0–100%; *p* = 0.001) respectively (Fig. 4). Notably, respecting the above-mentioned selection criteria for radiotherapy, even patients with severely impaired function at start of treatment had a reasonable chance for improvement. Seven patients with a visual acuity < 0.1 before treatment (mean 0.06 ± 0.02) showed a significant improvement to 0.43 ± 0.14, *p* = 0.04. Nine patients had visual field loss > 70% before radiotherapy (mean 84.7% ± 3.6%), which showed a non-significant reduction to 66.1% ± 10.4%, *p* = 0.11.

Changes in visual field loss and visual acuity showed a moderate correlation for the evaluated patients. Data was available for 24 patients, one additional patient was excluded due to complete blindness after radiotherapy (R^2^ = 0.41, Fig. 4). Ophthalmological outcome did not correlate with age, volume of PTV54 or EUD of PTV51 and PTV54 (data not shown). Patients with sheathlike growth of ONSM showed significantly greater reduction of visual field deficits compared to those with fusiform tumors (23.2% ± 5.6% vs. 2.1% ± 3.1%, *p* = 0.02).

### Long term outcome

**Fig. 5:**
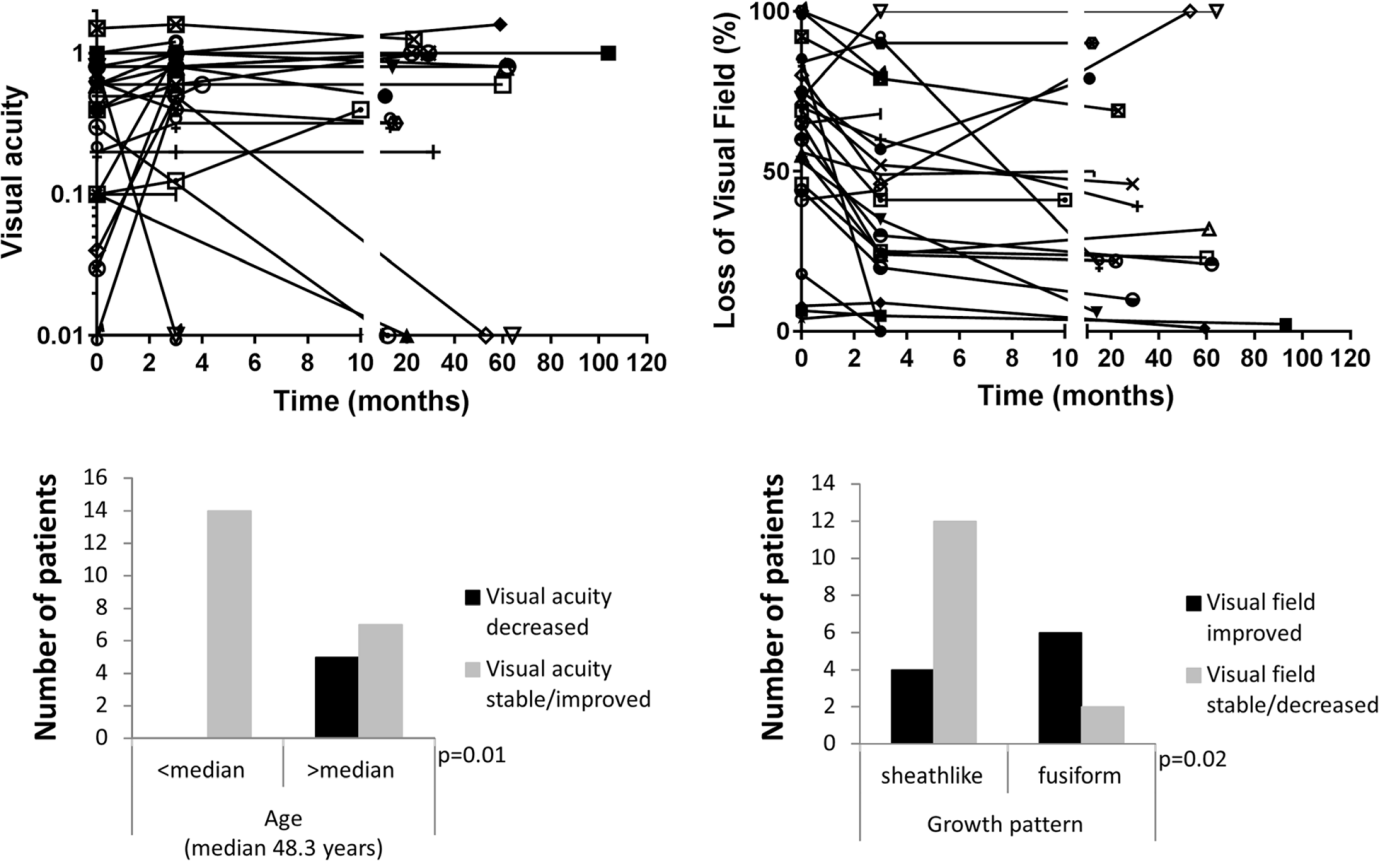
Long term visual outcome is plotted as visual acuity and loss of visual field over time for all patients individually. In total, five patients had severe loss of function of the treated eye over time. Visual field remained rather stable after initial treatment responses in most patients. All patients with decreased visual acuity over time after radiotherapy were treated at an age above the median age of the cohort. For visual field loss, a significant correlation was found between improved function and sheathlike tumor growth

No local progression was observed in follow-up MR imaging in any patient. Long term results of visual acuity and visual field loss were assessable for all patients in follow-up using the same method as for the first examination after radiotherapy. Results are shown in Fig. 5. Improved visual acuity was recorded for 9 patients, 12 patients showed stable function, in 5 patients visual acuity declined. Visual field was improved in 14 patients, stable in 8 patients and worsened in 2 patients (data missing for two patients). The two patients with increasing loss of visual field also had severely impaired visual acuity, thus the results in these patients might have been compromised by overall visual function. In total, 16 patients had improved overall visual function, 6 were stable and 4 patients declined (1 patient rated as stable with a decline in visual acuity and improved visual field).

Possible prognostic factors tested for the influence on long term results of visual function included age at start of radiotherapy (classified as above or below the median age of 48.3 years), size of PTV above or below the median of 4.1 cm^3^, sheathlike versus fusiform growth and involvement of the optic canal. For statistical analysis visual acuity and visual field was classified in decreased / stable versus improved and decreased versus stable / improved, respectively. The only factor significantly influencing decreased visual function was higher patient age at start of radiotherapy (Fig. 5). Improvement of visual field was significantly more frequent in patients with sheathlike tumor growth compared to fusiform tumors (Fig. 5). Of the five patients with a decline in visual acuity after treatment one had additional facial nerve palsy with corneal erosion, one had pre-existing localized systemic sclerosis. One patient showing typical signs of radiation induced optic neuropathy (RION) had pre-existing auto-antibodies to aquaporin 4. Steroid treatment did not improve visual function. Patients with decline of visual acuity are summarized and discussed in Table 3. In total, after interdisciplinary discussion, findings in two patients were classified as radiation toxicities (2/26, 8%). Interdisciplinary discussion involved additional diagnosis, ophthalmologic findings, time course between diagnosis, radiotherapy and decline in visual function as well as radiation dose distribution.

**Table 3:** Patients with worsened visual acuity

Clinical history		Discussion / presumable diagnosis
1	Male 48 years: Visual acuity 0.2 before and at the end of radiotherapy, perception of hand movements at next visit one year later. Optic atrophy. D2 optic nerve 53.41 Gy.	RION (Radiation-induced optic neuropathy)
2	Male 82 years: Two years before radiotherapy the patient suffered from a stroke with persisting facial nerve palsy on the side of the meningioma. Corneal ulcers were present before and after therapy. Visual acuity was perception of hand movements before radiotherapy and no light perception after therapy.	Multifactorial process including facial nerve palsy and corneal ulcers
3	Female 50 years. Visual acuity 0.63 6 weeks before radiotherapy. 2 months after radiotherapy only perception of handmovements. Unusual imaging finding: strong contrast enhancement even 5 years after therapy, relatively mild SSR analogon-uptake in PET.	Atypical PET-signal for ONSM before start of radiotherapy, possibility of misdiagnosis
4	Female 56 years. Visual acuity 0.04 1 week before radiotherapy. Relatively mild SSR analogon-uptake in PET. Visual acuity 0.5 3 months after therapy. Subacute visual loss accompanied by pain with eye movements 20 months after therapy. Visual loss to perception of hand movements, no improvement. At the time of visual loss a long and marked contrast enhancement was seen in the optic nerve which was much less pronounced 3 months earlier.	Atypical PET-signal for ONSM before start of radiotherapy, possibility of misdiagnosis
5	Female 70 years. Visual acuity 0.63 2 weeks before therapy. Six weeks after radiotherapy visual acuity was 0.16 and a macular oedema was seen in OCT. Visual acuity improved again to 0.4. Visual field improved. D2 eye 51.72 Gy	Radiation-induced retinopathy

## Discussion

The diagnosis and treatment of ONSM poses an interdisciplinary challenge for neuro-ophthalmologists and radiation oncologists. Patients often present with a long history of symptoms and misdiagnoses [30]. Anatomical imaging (CT, MRI) with high resolution might lead to the diagnosis in most cases [31]. However, for confirmation and to avoid surgical biopsies, the specificity of somatostatin-receptor-analoga PET imaging should be used [6]. After diagnosing ONSM interdisciplinary discussion of the management as described in Fig. 1 is crucial, as active surveillance might be equivalent to immediate treatment and spares the patients 6 weeks of radiotherapy and possible side effects [18].

Radiotherapy planning for high precision radiotherapy of ONSM has to take into account planning objectives which clearly prioritize sparing of OARs over ICRU-conformal coverage of PTV [32]. The main objective is not to threaten the functional outcome by limiting the dose at organs at risk. Dose coverage of the planning target volumes is of less concern as local control rates are excellent with the usual radiation dose of 50.0–54.0 Gy [19, 23, 26, 33]. At our institution, when starting Hyperion®-IMRT-based radiotherapy planning, plan quality increased with the treatment of the first ten patients and reached a plateau afterwards, indicating a learning curve for treatment planning.

The patient population selected for high precision radiotherapy showed a significant improvement in visual acuity and reduction of visual field defects at first neuro-ophthalmological follow-up after radiotherapy. Even in patients who showed severe deficits before radiotherapy the function improved early after treatment. A decline in visual acuity was observed in 5 / 26 patients (19%), which is in line with previous reports [25, 26]. Reports with lower rates of worsened visual function mostly also included asymptomatic patients with incidental imaging findings who would have undergone active surveillance following our treatment strategy [20]. Overall, radiotherapy is suitable to stabilize or increase visual function in the majority of patients, even with severe deficiency before start of treatment. Decline in visual acuity was only observed in patients aged older than the median age of our patient cohort. Tumors with sheathlike growth had a significantly higher chance of reduced visual field defects after treatment. The only published report about prognostic factors for functional improvement after high precision radiotherapy for ONSM identified no prior surgery and larger PTV as factors predicting a higher likelihood of improvement. However, comparison is limited by differences in the patient cohort with are a large number of patients with previous resection and diverse radiation schedules (25–66 Gy total dose in 1.8–5.0 Gy fractions) in this reported series [34].

A major concern is still patient selection. Because biopsy implies a high risk for the optic nerve alternative diagnostic measures are necessary. Somatostatin-receptor-analoga PET imaging offers a strong tool to select patients with the correct diagnosis [35]. Characteristics of the use of ^86^Gallium-DOTATOC-PET-CT have been reported by our group previously [36]. A closer look on the cases worsening in vision showed that two of them showed low somatostatin uptake but high gadolinium uptake. In the future we would consider this as a warning sign. Differential diagnosis for suspected ONSM includes granulomatous inflammation and other malignant diseases (metastases, lymphoma) [6, 37]. Most patients with ONSM have been treated with steroids usually under the diagnosis of optic neuritis prior to the indication for radiotherapy. If this has not been done we advocate a treatment attempt of at least one week with prednisolone 1 mg/kg or equivalent steroid dose.

## Conclusion

In summary, the treatment of ONSM requires close interaction between neuro-ophthalmologists and radiation oncologists and a stringent patient selection for radiotherapy. Optimal patient selection and timing of therapy start is the key to additional improvement in outcome. Treatment planning is sophisticated and relies on the experience of the planning team. Our study showed that experience in planning leads to better treatment parameters. Under these precautions, high precision radiotherapy for ONSM benefits most treated patients by stabilizing or even improving visual function.

## Data Availability

The datasets used during the current study are available from the corresponding author on reasonable request.

## Abbreviations

CT: Computed tomography
EQD2: Equivalent dose to 2 Gy fraction dose
EUD: Equivalent uniform dose
GTV: Gross tumor volume
ICRU: International Commission on Radiation Units and Measurements
IGRT: Image-guided radiotherapy
IMRT: Intensity modulated radiotherapy
MLC: Multileaf collimator
MRI: Magnetic resonance imaging
OAR: Organ at risk
OCT: Optical coherence tomography
ONSM: Optic nerve sheath meningioma
PET: Positron emission tomography
PTV: Planning target volume
RION: Radiation induced optic neuropathy
VMAT: Volumetric arc therapy
