# Correction to: Retrospective analysis of fractionated intensity-modulated radiotherapy (IMRT) in the interdisciplinary management of primary optic nerve sheath meningiomas

**DOI:** 10.1186/s13014-020-01706-0

**Published:** 2020-11-19

**Authors:** Franziska Eckert, Kerstin Clasen, Carina Kelbsch, Felix Tonagel, Benjamin Bender, Ghazaleh Tabatabai, Daniel Zips, Daniela Thorwarth, Bettina Frey, Gerd Becker, Helmut Wilhelm, Frank Paulsen

**Affiliations:** 1 Department of Radiation Oncology, Eberhard-Karls-University Tuebingen, Hoppe-Seyler-Str. 3, 72076 Tuebingen, Germany; 2 Centre for Neurooncology, Eberhard-Karls-University Tuebingen, Hoppe-Seyler-Str. 3, 72076 Tuebingen, Germany; 3 Department for Ophthalmology, Eberhard-Karls-University Tuebingen, Elfriede-Aulhorn-Str. 7, 72076 Tuebingen, Germany; 4 Department of Diagnostic and Interventional Neuroradiology, Eberhard-Karls-University Tuebingen, Hoppe-Seyler-Str. 3, 72076 Tuebingen, Germany; 5 Department of Radiation Oncology, Section for Biomedical Physics, Eberhard-Karls-University Tuebingen, Hoppe-Seyler-Str. 3, 72076 Tuebingen, Germany; 6 RadioChirurgicum, CyberKnife Suedwest, Klinik Am Eichert, Eichertstr. 3, 73035 Goeppingen, Germany

## Correction to: Radiation Oncology (2019) 14:240 https://doi.org/10.1186/s13014-019-1438-2

Following publication of the original article [1], the authors identified an error in Fig. 5. The correct Fig. 5 is provided below:

**Fig. 5:**
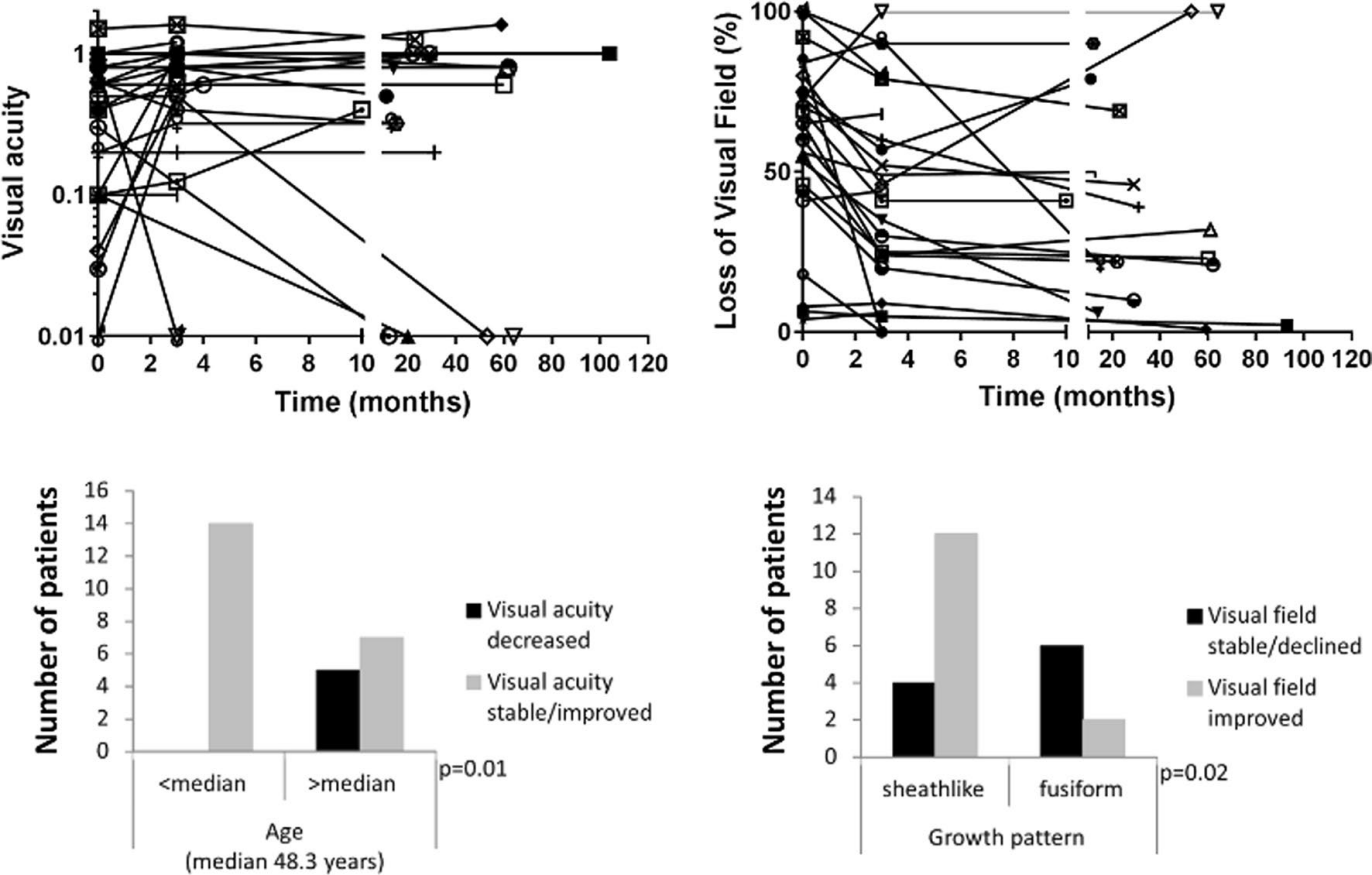
Long term visual outcome is plotted as visual acuity and loss of visual field over time for all patients individually. In total, five patients had severe loss of function of the treated eye over time. Visual field remained rather stable after initial treatment responses in most patients. All patients with decreased visual acuity over time after radiotherapy were treated at an age above the median age of the cohort. For visual field loss, a significant correlation was found between improved function and sheathlike tumor growth
